# Protease-Activated Receptor 1 as Therapeutic Target in Breast, Lung, and Ovarian Cancer: Pepducin Approach

**DOI:** 10.3390/ijms19082237

**Published:** 2018-07-31

**Authors:** Lidija Covic, Athan Kuliopulos

**Affiliations:** 1 Division of Hematology/Oncology, Tufts Medical Center, Boston, MA 02111, USA; athan.kuliopulos@tufts.edu; 2 Department of Medicine, Tufts Medical Center, Boston, MA 02111, USA; 3 Center for Hemostasis and Thrombosis Research, Tufts Medical Center, Boston, MA 02111, USA

**Keywords:** pepducin therapy, GPCRs, invasion, breast, lung and ovarian cancer, PAR1

## Abstract

The G-protein coupled receptors (GPCRs) belong to a large family of diverse receptors that are well recognized as pharmacological targets. However, very few of these receptors have been pursued as oncology drug targets. The Protease-activated receptor 1 (PAR1), which is a G-protein coupled receptor, has been shown to act as an oncogene and is an emerging anti-cancer drug target. In this paper, we provide an overview of PAR1’s biased signaling role in metastatic cancers of the breast, lungs, and ovaries and describe the development of PAR1 inhibitors that are currently in clinical use to treat acute coronary syndromes. PAR1 inhibitor PZ-128 is in a Phase II clinical trial and is being developed to prevent ischemic and thrombotic complication of patients undergoing cardiac catheterization. PZ-128 belongs to a new class of cell-penetrating, membrane-tethered peptides named pepducins that are based on the intracellular loops of receptors targeting the receptor G-protein interface. Application of PZ-128 as an anti-metastatic and anti-angiogenic therapeutic agent in breast, lung, and ovarian cancer is being reviewed.

## 1. Introduction

Cancer is the second most common cause of death in the US and worldwide and is exceeded only by heart disease. About 1.7 million new cancer cases are currently diagnosed in the US alone [1]. Reductions in smoking as well as improvements in early detection and treatment has shown a decline in a variety of cancers including lung and breast cancer. However, there is still poor survival outcomes in metastatic cancers including lung, breast, and ovarian cancers. The new and promising target Protease-activated receptor-1 (PAR1) is associated with solid tumor progression including primary growth, invasion, metastasis, and angiogenesis [2,3,4,5,6,7]. This review will focus on its functional role and evaluate PAR1 as a potential therapeutic target in breast, lung, and ovarian cancer. We will discuss the role of PAR1-biased signaling of the proteolytic action of canonical (thrombin) and non-canonical (collagenase matrix metalloprotease 1 (MMP-1)) [2,8]. Our laboratories have developed an approach to study receptor-mediated G protein activation using palmitoylated peptides, which are also known as pepducin technology that act as allosteric modulators of GPCR-dependent G-protein signaling [9,10,11]. PAR1 derived pepducin PZ-128 is in a Phase II clinical trial for thrombotic indication [11,12,13]. The effects of PZ-128 in multiple cancer preclinical studies will be discussed.

## 2. PAR1 Structure, Function, and Downstream Interactors

### 2.1. PAR1 Structure and Protease Activation

**Figure 1:**
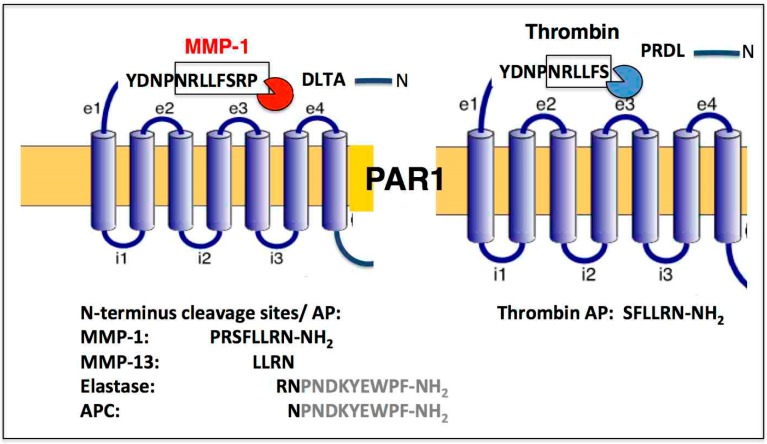
PAR1 biased signaling is orchestrated by a diverse set of unique proteases, both noncanonical (MMP-1) and canonical (thrombin). PAR1 activation by MMP-1 and thrombin results in two different cleavage sites with the MMP-1 generated activating peptide (AP) PR-SFLLRN-NH_2_ and the thrombin generated SFLLRN-NH_2_. N-terminal cleavage sites are listed for MMP-13, Elastase, and APC. Tethered AP sequences are shown for Elastase and APC.

PAR1 is a family member of the four PARs that also include PAR2–4, which act as exquisite sensors to a select group of proteases [14]. PAR1 is classically activated through thrombin [15] by cleaving its exodomain at the R_41_–S_42_ peptide bond (Figure 1) [15,16,17]. Matrix metalloproteases (MMPs) are well known as important mediators of both atherothrombotic disease [18,19] and cancer [2,20,21,22,23]. This is especially relevant for the metastatic processes since physiologic levels of *MMPs* are low, but expression is elevated in most invasive cancers [2,18,24].

PAR1 expression is increased in a number of cancers including breast, colon, and lung cancer. A study using a xenograft model of breast carcinoma cells originally demonstrated a critical role for MMP-1 derived from tumor-infiltrating fibroblasts in the cleavage of PAR-1, which appears to drive cancer cell migration and invasive behavior of the tumor [2]. This finding led to a change in the central dogma that PAR1 can be cleaved at a divergent site from thrombin and activated by specific proteolysis by noncanonical MMP-1 at LD_49_^P_50_RSFL (Figure 1). PAR1 is also activated by murine MMP-1, namely MMP-1a [21,22,23,25], and by another collagenase MMP-13 [19,26,27]. The role of MMP-13 in PAR1-mediated activation in cancer has not been well studied except in context of tumor cell expression [28,29]. 

MMP-13 was identified as having the capacity to cleave and activate PAR1 on cardiac fibroblasts and cardiomyocytes, which results in pathologic activation of downstream signaling events that contribute to heart failure [27]. Other noncanonical protease agonist of PAR1 such as APC, plasmin, factor Xa, and elastase are important in diverse physiological non-cancer processes [16,30,31].

In particular, collagenase MMP-1 that can degrade type I, II, and III collagens is highly expressed and metastatic in breast cancer [32]. The levels of MMP-1 in tumor samples of node positive patients were significantly higher than in samples of node negative patients and are associated with the 1G/2G polymorphism of the promoter region of MMP-1 in breast cancer patients [33]. Moreover, MMP1-PAR1 activation induced secretion of several angiogenic factors from ovarian carcinoma cells including IL-8, GRO-α, and MCP-1 [7]. In addition, another important role of MMPs is the proteolytic alteration of chemokines [34].

Furthermore, MMP-14 and MMP-2 proteolytically [35] activate TGF-β which is a major tumor promoting factor that leads to increased invasion and metastasis. However, MMPs can generate both angiogenesis promoting and inhibiting signals depending on the cleavage product of the diverse MMPs [36]. MMP-9 and the angiogenesis vascular endothelial growth factor (VEGF) bioavailability switch promote angiogenesis. In contrast, a cleavage of collagen by MMP-3, -7, -9, -13 or -20 can generate biologically active angiostatin, which is a suppressor of angiogenesis.

PAR1 is a member of the family A of GPCRs that belongs to the larger delta subfamily and is most similar to the purinergic, the olfactory, and the glycoprotein receptors. Zheng et al. [37] provided a high 2.2 Å resolution crystal structure of human PAR1 complexed with a PAR1 antagonist, vorapaxar, which was found to bind close to the extracellular surface of PAR1 over a large surface area. The vorapaxar binding pocket in PAR1 resembles rhodopsin and sphingosine-1-phospahate receptor ligand sites. In PAR1, residue D367 forms a strong hydrogen bond that results in displacing the cytoplasmic end of TM7 that is displaced inward towards TM2, which is a position similar to the active conformation of the β2-adrenergic receptor bound to heterotrimeric G protein. Substitution analysis of PAR1 residues and the NMR structure of the extracellular domain provided the bases for the hypothesis that the tethered peptide may first bind, superficially to the extracellular domains as a multistep sequential mechanism before binding more deeply into the core through conformational intermediates. We have generated a lipidated peptide derived from the entire PAR1third intracellular (i3)-loop known as P1pal-19 [38] that can allosterically activate the PAR1 receptor through the TM7 D/NPxxYYY motif through a dimer-like mechanism between the i3 loop and the eight helix [39]. Our early work identified the eighth helix as critical including cysteine palmitoylation and interactions with the adjacent NPXXY motif (TM7) and intracellular loop-1 (i1), which has an important role in the transfer of the signal from PAR1 to the G-protein [40]. Based on the crystal structures of the on and off-states, the D/NPxxYYY containing region undergoes a major structural rearrangement. D367 and Y371 form hydrogen bonds with TM2 and TM1 residues in PAR1, which was suggested previously to be similar to the active β2-adrenergic receptor activation mechanism.

### 2.2. Diverse Downstream Interactors: Canonical and Non-Canonical Role and Signaling

**Figure 2:**
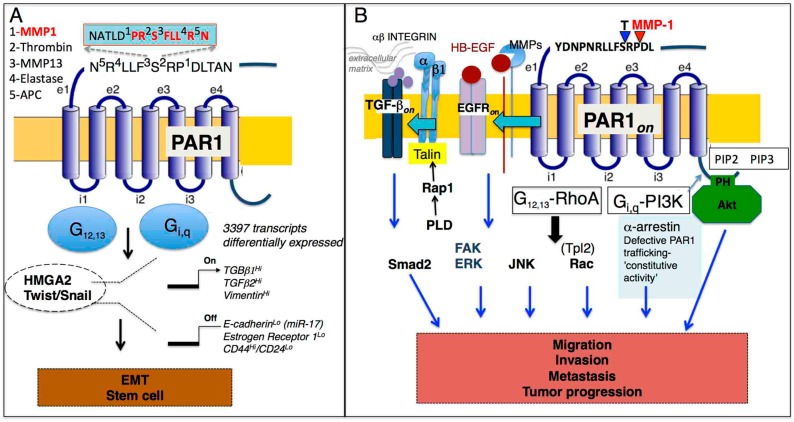
(**A**). Schematic diagram of PAR1 and HMGA2, Twist/Snail mediated transcriptional regulation of mesenchymal markers, and down-regulation of epithelial markers. Both HMGA2 and Twist further regulate expression of PAR1. TGF-β has a prominent role in the regulation of these processes and stem cell markers. PAR1-biased signaling is orchestrated by a diverse set of unique proteases canonical (thrombin) and noncanonical (MMP-1, MMP-13, elastase, APC) that cleave the PAR1 extracellular loop 1 (e1) at indicated amino acid residues. (**B**) Multiple signaling pathways control PAR1-mediated tumor progression (migration, invasion, and metastasis) that are mediated through PAR1 coupled G-proteins including G_12,13_ and G_i,q_. PAR1 is constitutively turned on as a result of defective PAR1 trafficking. Parallel PAR1 signaling pathways through G_12,13_-RhoA link outside extracellular matrix signals with β1 integrin, TGF-β, and EGFR transactivation that regulate proliferative and migratory responses (ERK1/2, FAK, Smad2) while G_i,q_-PI3K harness Akt-survival pathways and Rac result in the invasion and, ultimately, in metastasis.

PAR1 is activated differentially by distinct canonical (thrombin) and noncanonical (MMP-1) generated tethered ligands within the extracellular N-terminal domain that result in subtle differences in the allosteric mechanisms of activation and transmembrane signaling to a variety of unique downstream integrators (Figure 2). These diverse signaling pathways control the Epithelial-to-Mesenchymal Transition (EMT) and may be related to stem cell biology, tumor progression (migration/invasion/metastasis), angiogenesis, and barrier function (Table 1). We will discuss the implications of protease-biased signaling of PAR1 in these critical processes as related to cancer.

#### 2.2.1. Role of PAR1 in the Epithelial-to-Mesenchymal Transition (EMT)-Stem Cell

Our work showed that PAR1 ectopic expression leads to cancer stem cell growth associated with an increase in the CD44^high^/CD24^low^ ratio, self-renewal, and anchorage independent growth in breast cancer (Table 1) [41]. Multiple examples of up-regulated PAR1 expression in diverse tumors have also been previously reported [5,52,53]. Atopic expression of PAR1 in the MCF7 breast cancer cell line was examined for resultant global changes in gene transcription using the Human Genome U133A 2.0 Array (Affymetrix, Bedford, MA, USA) containing 14,500 human transcripts. Overall, there was a dramatic transcriptional shift in 3397 transcripts with an upregulated expression of mesenchymal markers including vimentin and down-regulation of epithelial markers including E-cadherin as well as the estrogen receptor (Figure 2A). Both canonical and noncanonical activation of PAR1 resulted in direct regulation of vimentin expression. We also found that vimentin expression increased along with the histologic grade of the tumor in breast cancers. In contrast, there was no vimentin expression detected in normal tissue. This remarkable transcriptional shift was highly indicative of a mesenchymal transition. There was an increase in the expression of TGF-β family members that have been well-established as potent inducers of mesenchymal transition in mammary cells that involved acquisition of tumor stem-like properties. E-cadherin was found to be regulated by miR-17 through the NF-κB pathway [42].

The most widely studied EMT regulators include Snail and Twist and either of these transcription factors can induce EMT [43,54,55,56]. Our work found that PAR1 induced a basal-like phenotype in human breast cancer cell lines through the non-histone chromatin-binding protein and a high-mobility-group non-histone chromosomal protein called HMGA2 [57]. HMGA2 has been identified as an essential driver in a variety of cancers [58,59,60]. The TGF-β signaling pathway, in particular TGF-β1, was highly induced in PAR1 expressing cells and was previously identified as an important regulator of HMGA2 [61]. The regulatory role of HMGA2 and PAR1/TGF-β still remains elusive and is currently being investigated.

#### 2.2.2. PAR1 and Migration/Invasion/Metastasis/Tumor Progression

Our previous work identified noncanonical MMP-1 as a protease that cleaved and activated PAR1 [2,18]. MMP-1-PAR1 signaling activated survival pathways in breast cancer cells similar to thrombin with distinct kinetics of duration [4]. We also demonstrated that, in some breast cancers, tumor-stromal interactions play a critical role [2,20] and that stromal-derived MMP-1 can drive tumor progression, invasion, and metastasis through the activation of PAR1. Both MMP-1 expression and collagenase activity is upregulated in breast tumors carrying PAR1 as compared to normal mammary pads. The high PAR1 expressing cells showed a dose-response in their ability to proliferate that was directly related to PAR1 surface expression. Akt played a prominent role in cell growth, survival, and proliferation. Persistent activation of Akt was found using both PAR1-expressing breast cancer cells in in vitro and in vivo systems. Persistent MMP1-PAR1-Akt signaling resulted in the metastasis of breast tumors to the lung. Consistent with our findings, work by Bar-Shavit and colleagues identified an association between Akt and PAR1 via the pleckstrin homology (PH) domain in PAR1 [44], which is important in the tumor growth and trophoblast invasion processes that are also lipid (PIP2 and PIP3) driven. Furthermore, biased PAR1 signaling and PAR1-activation with elastase cleaved PAR1 at L_45_^R_46_, which also resulted in the PH-Akt association and MAPK activation. However, it still remains unclear how the biased PAR1 signaling activated by multiple canonical and noncanonical downstream pathways differentially regulates tumor progression and warrants further investigation.

Constitutive PAR1 signaling may be a consequence due to a loss of tumor suppressor α-arrestin (ARRDC3) and deregulated PAR1 trafficking [45,62]. Another report of the tumor suppressor called ALEX1 in gastric cancer inhibited thrombin-induced metastasis through Rho GTPase and Rac activation [46]. Thrombin mediated PAR1 signaling is regulated through the tumor progression locus 2 (Tpl2) by activating Rac1 and focal adhesion kinase (FAK), which promoted migration through actin cytoskeleton reorganization [47]. Tpl2 ablation down-regulated PAR1 mediated activation of a variety of proinflammatory and proangiogenic factors including *MMPs*, *VEGF*, *Cyr61* and *SDF1α*. In addition to the Tpl2 mediated response, there are reports of thrombin mediated PAR1-G_12/13_ RhoA activation of phospholipase D (PLD) that, in turn, activated Rap1 in 1321N1 and U373MG glioblastoma cells, which is a well-known Ras-GTPase readily activated in platelets [48]. Analogous of the platelet mechanism of activation, Rap1 activated the β1 integrin pathway that led to an increase in FAK and ERK1/2 phosphorylation [63]. A schematic illustration in Figure 2B summarizes G_12,13_-RhoA and G_i,q_-PI3K-mediated activation of Akt, JNK, Tpl2-Rac, and PLD-Rap1-β1 integrin dependent FAK, ERK activation. These PAR1-dependent multiple signaling cascades regulate complex processes of tumor progression that include migration and invasion that ultimately leads to metastasis, which makes PAR1 an attractive and important therapeutic target.

The EGFR transactivation mediates PAR1 and its implications in tumor progression (Figure 2B). Previous reports suggest that proteolytic activation of PAR1 by thrombin-dependent MMP activity resulted in the persistent activation of EGFR and ErbB2/HER2 through extracellular signal-regulated kinase-1 and kinase-2 (ERK1/2) in invasive breast carcinoma [64,65]. The MMP mediated shedding of HB-EGF may be induced by ROS that is dependent on the plasma membrane-associated NADPH oxidase (Nox) family members. In particular, p47^phox^ phosphorylation and NADPH oxidase activation generate a reactive oxygen species (ROS) through O_2_ by using NADPH as an electron donor [66]. For PAR1, thrombin induction resulted in the activation of Rho/ROCK mediated TGF-β activation that, in turn, activated Smad2 complexes [65].

#### 2.2.3. PAR1 and Angiogenesis Barrier Function/Dissemination

Angiogenic factors have been well recognized as important contributors toward disseminated tumor growth and metastasis. Multiple studies in breast, ovarian, and lung cancer provide convincing evidence for the role of PAR1 in regulating blood vessel formation and expression of angiogentic factors such as VEGF, IL-8, and GRO-α [2,5,7]. In ovarian cancer, noncanonical MMP1-medited PAR1 activation was critical in regulating chemokine signaling. The role of MMP-1 in highly metastatic human epidermoid carcinoma, HEp3, was shown to be PAR1-dependent and shown to be an important regulator of vascular permeability, tumor cell intravasation, and metastatic dissemination [50] in agreement with our data in vascular integrity [67,68]. Other reports identified thrombin as a regulator of barrier function and a pro-inflammatory and pro-angiogenic mediator through the regulation of Ca^2+^ [49]. Likewise, prothrombin was found to be a major determinant of the metastatic progression of colon cancer cells [51].

## 3. PAR1 in Cancer Therapeutics

The pepducin approach is a process to modulate G-protein coupled receptors (GPCRs) is by targeting the intracellular faces of GPCR receptors through pepducin technology [9,10,39,69,70,71,72,73,74,75,76,77,78]. Pepducins are cell-penetrating lipidated peptides designed to target the intracellular (i1–4) loops of the receptor. The pepducin PZ-128, also known as P1pal7 [9,12,13,79] targets PAR1 and has been extensively validated and advanced into the clinical setting with completion of a Phase I clinical trial (*n* = 34 subjects) and a nearly completed Phase II (*n* = 100) multicenter, randomized, placebo-controlled clinical trial to treat severe cardiovascular disease in patients undergoing percutaneous coronary interventions. Below, we will review preclinical data in experimental mouse models of breast, lung, and ovarian cancer that validate PZ-128 and the PAR1 target in these tumors.

### 3.1. PZ-128 and Breast Cancer Preclincal Studies

Work on PAR1 as an important clinical target with a critical multi-role in thrombosis, inflammation, and vascular biology has been investigated by multiple laboratories since the PAR1 discovery in 1991 as a thrombin receptor [15]. However, under pathophysiological conditions in carcinomas, PAR1 is an oncogenic protein, which is a potent inducer of cancer cell migration, invasion, survival, and metastasis [3,4,80,81,82,83,84]. Our laboratory evaluated PAR1-derived PZ-128 as a potential PAR1 inhibitor to suppress breast cancer progression. The efficacy models are summarized in Table 2. PAR1 was atopically expressed in PAR1-null, estrogen-sensitive MCF-7 cells and tested for its ability to promote tumor-growth and invasion in nude mice. PAR1 expression resulted in a 100% rate of tumor formation while PAR1-null MCF-7 cells did not form any palpable tumors (Table 2) [2]. These PAR1-driven tumors could be significantly inhibited (62%, *p* < 0.01) with PZ-128. PAR1-dependent tumor growth was MMP1-mediated because treatment with MMP-1 Inh resulted in 82% inhibition of tumor growth. Tumors were examined for vascularity using von Willebrand Factor. The mean (±2 SEM) blood vessel density was 10.4 ± 2.2 for the untreated group, 2.6 ± 1.0 (*p* = 0.002) for the PZ-128-treated group, and 3.6 ± 1.5 (*p* = 0.006) for the MMP-1 Inh treated group. Therefore, both inhibition of PAR1 and MMP-1 resulted in the inhibition of angiogenesis. Likewise, PAR1 expressing MCF-7 cells could form metastatic lesions in lungs with a 100% penetrance rate similar to the highly metastatic MDA-MB-231 while the PAR1-mutant R310E that lacked signaling abilities had undetectable metastatic lesions in the lung [41]. Most strikingly, there was a similar reduction in the number of metastatic lesions with either treatment as monotherapy, PZ-128, or MMP-1 Inh (75% and 88%, respectively, *p* < 0.001) [4].

We assessed the efficacy of PZ-128 in a mammary fat pad model with highly invasive, estrogen-independent PAR1-expressing MDA-MB-231 cells. Since Docetaxel (Taxotere) is a chemotherapy drug used to treat breast cancer that has become the standard of care for preoperative neoadjuvant, adjuvant, and metastatic settings [85], it was tested in combination with PZ-128 for efficacy using MDA-MB-231 cells. Dual treatment with Taxotere (10 mg/kg) and PZ-128 (10 mg/kg) was administered early (day 2) and resulted in near complete (98%) (*p* < 0.05) inhibition of tumor growth. In contrast, monotherapy with either Taxotere or PZ-128 had no effect when compared to the vehicle-treated control [4]. Delayed treatment (day 15) with dual therapy still significantly inhibited (60%, *p* > 0.01) tumor growth. Upon sectioning, the tumors were stained for apoptosis using the TUNEL process, which identified on average 60% of apoptotic regions within tumors treated with dual therapy.

The Akt survival pathway was identified as an important mechanism of MMP1-PAR1 signaling. Biochemical analysis of MDA-MB-231 derived tumors 200 mm^3^ in size before the start of five-day treatment with PZ-128 (10 mg/kg) or MMP1 Inh (5 mg/kg) together with a single dose of Taxotere (10 mg/kg) were carried out. There was a 54% and 61%, (*p* < 0.05), respectively, reduction in Akt phosphorylation in tumors with dual treatments. Therefore, pAKT could be potentially used as a direct biomarker for the efficacy of PZ-128 in a clinical setting in the treatment of breast cancer. Taken together, these data provide strong evidence that blockade of PAR1 with PZ-128 and Taxotere could be beneficial in advanced, metastatic breast cancer.

### 3.2. Efficacy of PZ-128 and Role of MMP-1a in Lung Cancer

PAR1 is a poor prognostic marker in lung cancer that correlates with reduced survival in non-small-cell lung cancer (NSCLC) [86]. In 2007, bevacizumab (Avastin) in combination with carboplatin and paclitaxel was approved as the first-line treatment of patients with unresectable, locally advanced, recurrent or metastatic non-squamous NSCLC [87]. We carried a comparative efficacy study of PZ-128 versus Avastin in a well-established A549 xenograft model of lung adenocarcinoma injected subcutaneously in the flank of the mouse. PZ-128 and Avastin significantly inhibited tumor growth (*p* < 0.01) with 75% and 67% inhibition, respectively (Table 2, [5]). We also showed that PAR1 can regulate VEGF production through the ERK1/2-dependent signaling pathway. Therefore, patients with metastatic NSCLC may benefit from the ERK1/2 blockade. Further studies are needed in order to determine if PZ-128 could induce a therapeutic response.

The role of MMP-1 in NSCLC is not well understood. In order to address the role of mouse homolog MMP-1a, knockout mice were generated (*MMP1a*-KO) [23]. Lewis lung carcinoma (LLC1) is a highly tumorigenic cell line that expresses high levels of PAR1. Mouse LLC1 cells were implanted subcutaneously into the abdominal fat pad of *MMP1a*-KO and wild type (WT) C57CL/6 mice. The progression of tumor growth was followed over 26 days. The loss of stromal MMP1a resulted in a 50% reduction of tumor growth when compared to WT-MMP1a mice (*p* < 0.001). There was a significant decrease (30%) (*p* < 0.01) in angiogenesis in the *Mmp1a*-KO mice when compared to wild-type mice.

In order to verify that MMP-1a tumor growth was PAR1-mediated in LLC1 cells, PAR1 was stably silent due to using a short hairpin-RNA (shPAR1) or control (shLuci). Consistent with our hypothesis, LLC1 (shPAR1) lagged in growth and formed smaller tumors (31% inhibition, *p* < 0.001) when compared to LLC1 (shLuci). Together these data provide support for the role of a mouse homolog of human MMP-1, namely MMP-1a in activation of PAR1.

### 3.3. PZ-128 as a Therapeutic Target in Ovarian Cancer

Ovarian cancer is still one of the deadliest gynecologic malignancy because of an early metastatic spread into the abdominal cavity. Despite some advances, a taxane such as Docetaxel still remains the standard-of-care in combination with a platinum compounds (cisplatin or carboplatin). A murine metastatic model of intraperitoneal (i.p.) ovarian cancer was conducted by injecting extremely aggressive and metastatic OVCAR-4 cells into an intraperitoneal cavity. The treatment regimen was PZ-128 (3.2 mg/kg i.p. every other day) or MMP-1 Inh (5 mg/kg) in combination with Docetaxol (10 mg/kg i.p. once weekly). Metastatic progression was determined using a histological staging system that consisted of examining metastatic progression through omentum and invading through the diaphragm with metastasis to the lung and heart. All untreated (vehicle treated) mice had full-blown metastasis through the diaphragm and into the lungs and mediastinum (Table 2) [6]. In contrast, dual therapy with PZ-128 prevented invasion across the diaphragm into a thoracic cavity (*p* = 0.01) relative to docetaxol alone. Similar results were observed with MMP-1 Inh (*p* = 0.003). These data strongly support an MMP1-PAR1 signaling pathway as an important target for preventing the metastatic spread in ovarian cancer. There was a significant reduction (73% to 92%) in blood vessel formation with dual PZ-128 and dual MMP-1 Inhibitor therapy as compared to docetaxol alone. The mean (±2 SEM) blood vessel density was 13.7 ± 1.5 for the vehicle treated group, 8.1 ± 2.2 for the docetaxel group, 2.2 ± 0.4 (*p* < 0.005) for the PZ-128-treated group, 2.2 ± 0.4 (*p* < 0.005) for the dual PZ-128 + docetaxel group, and 3.0 ± 0.5 (*p* < 0.005) for the MMP-1 Inh + docetaxel treated group. Therefore, monotherapy with PZ-128 and MMP-1 Inh significantly inhibited blood vessel formation.

Ovarian peritoneal carcinomas produce large volumes of ascitic fluid. Using OVCAR-4 and SKOV-3 peritoneal carcinoma models, there was significant inhibition (60%, *p* = 0.0017) of ascitic fluid accumulation using PZ-128 as monotherapy (10 mg/kg every second day) when compared to vehicle control in an OVCAR-4 peritoneal carcinoma model. Similar results were seen with SKOV-3 cells. This effect on ascites accumulation may be a direct effect of an MMP1-PAR1 dependent endothelial barrier function. Taken together, PZ-128 is an excellent therapeutic for ovarian cancer treatment.

## 4. Pepducin Technology and Anti-Cancer Therapy: Future Prospective

PAR1 is a well-known target on human platelets that may benefit high-risk patients with a history of myocardial infraction (MI) or peripheral arterial disease (PAD) and reduce thrombotic events. Vorapaxar, atopaxar, and PZ-128 are the three PAR-1 antiplatelet agents that have undergone clinical development to various stages (Table 3). Vorapaxar is the first PAR-1 antagonist that was approved by the US Food and Drug Administration (in US and Europe) in May 2014 to reduce the risk of MI, stroke, and cardiovascular death and for the revascularization in patients with a previous MI or PAD (US). Vorapaxar is counter-indicated in patients with a history of prior stroke or transient ischemic attack (TIA). Since antiplatelet therapy is given on top of two other drugs (aspirin and P2Y12 inhibitors), there was a risk of increased bleeding with both vorapaxar and atopaxar. The use of the approved drug varapaxar as an anti-platelet agent is not clear due to safety concerns. However, this is less likely with PZ-128 because it is only given as a single IV dose to coronary artery disease (CAD) and acute coronary syndrome (ACS) patients during PCI. Currently, PZ-128 is well positioned as a promising antiplatelet agent that showed rapid platelet PAR1 inhibition appearing at 15 min which is faster than the P2Y12 oral drug clopidogrel (Plavix) used as the standard of care for patients undergoing percutaneous coronary intervention (PCI). PZ-128 has a plasma half-life of about 2 h and a pharmacodynamic half-life of about 24 h with 65–100% inhibition of PAR1 activity by 30 min [13]. There were no effects on bleeding, coagulation, clinical chemistry, or electrocardiography (ECG) parameters. The PZ-128 inhibitory effect was fully reversible with saturating concentrations of the PAR1 agonist.

The question remains whether PZ-128 or other currently developed PAR1 drugs could be used for the treatment of metastatic breast, lung, or ovarian cancers. PAR1-biased signaling is orchestrated by a diverse set of unique proteases canonical (thrombin) and noncanonical (MMP-1, MMP-13, elastase, APC) that cleave PAR1 and may stabilize diverse allosteric conformations that are perfectly tuned to activate diverse down-stream signaling (Figure 2). Therefore, the pepducin approach could provide an advantage because it could inhibit diverse allosteric conformations at the receptor-G-protein interface. Direct pharmacologic comparative studies in relevant cancer cell lines between vorapaxar, atopaxar, PZ-128 and other PAR1-derived pepducins using a set of proteases agonists such as thrombin, MMP-1, MMP-13 and elastase could address this question. Pepducin technology can readily target other parts of the receptor-G-protein interface to lead to design of a biased antagonists with favorable pharmacologies.

In summary, we provide evidence for the PAR1 inhibitor PZ-128 as an anti-metastatic and anti-angiogenic inhibitor in breast, lung, and ovarian cancer murine models and other relevant systems. We show synergy between deoxycycline (the standard-of-care) and PZ-128 as a therapeutic approach in breast and ovarian cancers. Furthermore, our results provide a mechanistic rationale for the use of a PAR1 inhibitor for breast cancer therapy and the employment of Akt and HMGA2 as potential biomarkers for efficacy.

## Figures and Tables

**Table 1:** Summary of PAR1-medicated signaling pathways that regulate Epithelial-to-Mesenchymal Transition (EMT), tumor progression, and angiogenesis/barrier function in solid tumors.

PAR1	Canonical/Non-Canonical	Signaling Pathways	Function	Ref
Breast Cancer	Canonical/noncanonical	HMGA2/EMT	EMT/metastasis	[41]
Breast Cancer		NF-κB/miR-17/E-cadherin	EMT	[42]
Cancer Stem Cell		Twist/Hippo pathway/TAZ	EMT	[43]
Breast Cancer	Noncanonical	Akt/E-cadherin/vimentin	Metastasis	[4]
Breast Cancer	Canonical	plekstrin homology (PH)-Akt/Etk/Bmx/Vav3	Cell invasion	[44]
Basal-like breast carcinoma		α-arrestin (ARRDC3)/JNK	Cell invasion	[45]
Gastric cancer		ALEX1/Rho GTPase	Tumor progression	[46]
Breast Cancer	Canonical	Tpl2/Rac1/FAK	Migration	[47]
U373MG glioblastoma	Canonical	PLD/RhoA/Rap1A/β1 integrin/FAK/ERK	Proliferation, tumor growth	[48]
Breast Cancer	Noncanonical			
Lung Cancer		VEGF	Angiogenesis	[5]
Ovarian cancer	Noncanonical	CXCR1/2	Angiogenesis	[7]
Endothelial cells	Canonical	Ca^2+/^Na^+^/Ca^2+^ exchange/ROS/ERK1,2	Angiogenesis/barrier function	[49]
HEp3 epidermoid carcinoma	Noncanonical	Tumor MMP-1	Dissemination/vascular permeability metastatic	[50]
Colon Cancer	Canonical	fibrinogen, stromal PAR1	Disseminated growth	[51]

**Table 2:** Summary of preclinical studies carried out with PZ-128 and the MMP-1 Inhibitor in breast, lung, and ovarian cancer.

Cell	Tumor Model	Type of Assay	Outcome	Ref
*Breast Cancer*				
MCF7-PAR1 (clone N55)	Mammary fat pad NCR nu/nu mice	xenograft model (tumor size)	>PAR1-tumor incidence 100% PAR1-null 0% after 6 weeks	[2]
		xenograft model (tumor size)	>PZ-128 (10 mg/kg) inhibits 62% MMP-1 Inh (5 mg/kg) inhibits 82% of tumor growth	
		histology vWF	>65% to 75% inhibition in angiogenesis	[2]
MDA-MB-231	Mammary fat pad NCR nu/nu mice	xenograft model (tumor size)	>dual treatment with PZ-128/Taxotere (Docetaxel) treatment day 2 98% inhibition	[4]
		xenograft model (tumor size) TUNEL staining	>delayed dual treatment day 15 60% inhibition of tumor growth, 60% apoptotic area	[4]
MDA-MB-231	Mammary fat pad NCR nu/nu mice	Western blot	>pAkt as biomarker inhibited 54% by PZ-128 after 5-day treatment MMP-1 Inh 5-day treatment 61% Inhibition	[4]
MDA-MB-231/GFP	Metastasis to lung NCR nu/nu mice Tail vein	lung histology	>reduction in metastatic incidence by PZ-128 75% MMP-1 Inh 88% inhibition	[4]
MCF7-PAR1 (clone N55)	Metastasis to lung NCR nu/nu mice Tail vein	lung histology	>PAR1-tumor incidence 100%	[41]
MDA-MB-231	Metastasis to lung NCR nu/nu mice Tail vein	lung histology	MDA-MB-231 tumor incidence 100%	[41]
MCF7-PAR1 (R310E)	Metastasis to lung NCR nu/nu mice Tail vein	lung histology	R310E tumor incidence 0%	[41]
*Lung Cancer*				
A549 NCR nu/nu mice	subcutaneous	xenograft model tumor size	>75% inhibition of tumor growth PZ-128 monotherapy (10 mg/kg) Compared to Avastin (5 mg/kg) 67% inhibition of tumor growth	[5]
LLC1 *MMP1a*-KO mice C57Bl (WT) mice	subcutaneous	xenograft model tumor size histology vWF	>LLC1 tumor growth in *MMP1a*-KO mice vs WT have 50% inhibition 30% inhibition in angiogenesis	[21,22,23]
LLC1-shPAR1 LLC1-shControl	subcutaneous	xenograft model tumor size	>31% inhibition in tumor growth with shPAR1	[23]
*Ovarian Cancer*				
OVCAR-4	Intraperitoneal cavity	Endothelial barrier	>PZ-128 (10 mg/kg i.p. every other day) reduced ascites formation by 60%	[6]
SKOV-3	Intraperitoneal cavity	Endothelial barrier	reduced ascites formation by 60%	[6]
OVCAR-4	Peritoneal carcinomatosis	Histology	>84% to 96% inhibition of angiogenesis monotherapy with MMP1 Inh and PZ-128	[6]
OVCAR-4	Peritoneal carcinomatosis	Histology	>dual treatment with PZ-128 or MMP-1 Inh/Taxotere (Docetaxel) inhibition of metastatic progression through diaphragm and thoracic cavity	[6]

**Table 3:** Vorapaxar, atopaxar, and PZ-128 are the three PAR-1 antiplatelet agents that have undergone clinical development.

PAR1 Drug	Clinical Trials	Indication	Results	Adverse Events
**Vorapaxar (Zontivity)SCH 530348**	Phase 3 [88,89,90] TRA 2P-TIMI50 [89] (*n* = 26,499) TRACER (*n* = 12,944)	Coronary Artery Disease (patients with high risk of ischemic events)	Significantly reduced the occurrence of the primary endpoint, CV death, MI, or stroke	Increased risk of intracranial hemorrhage (ICH)
**Atopaxar** **E5555**	Phase 2 LANCELOT-ACS [91] *n* = 603 J-LANCELOT-ACS [92] (*n* = 241) LANCELOT-CAD [93] (*n* = 720) J-LANCELOT-CAD [94] (*n* = 263)	Coronary Artery Disease	Increased bleeding (TIMI classification)	Transient elevation in liver transaminases and dose-dependent QTc prolongation without apparent complications
**PZ-128**	Phase 1 [13] *n* = 32; Phase 2 (NCT02561000) TRIP-PCI *n* = 100	Coronary Artery Disease	Anti-platelet effect no effects on bleeding, coagulation, clinical chemistry, or ECG parameters	Drug is well tolerated during IV infusion at therapeutic doses of 0.5 mg/kg

## Abbreviations

Canonical	a thrombin mediated activation of PAR1
Noncanonical	protease that is distinct from thrombin that generates a unique tethered ligand sequence in PAR1
HMGA2	High-mobility group AT-hook2 nuclear protein-a member of the superfamily of non-histone chromatin family that regulate gene expression through remodeling of the chromatin
EMT	epithelial-mesenchymal transition
NF-κB	nuclear factor-kappaB
miR-17	microRNA
E-cadherin	type-1 transmembrane glycoprotein
Twist	basic helix-loop-helix transcription factor
Hippo pathway	kinase cascade especially important in development
TAZ	transcription regulator 1
Akt	protein kinase B
Vimentin	structural protein
PH	plekstrin homology
Etk	tyrosine kinase
ARRDC3	Arrestin domain containing 3β-arrestin protein
JNK	Jun-N-terminal kinase
ALEX1	a tumor-suppressor member of the armadillo family
Tpl2	tumor progression locus 2 kinase
Rac1	Rho GTPase
PLD	phospholipase D
RhoA,Rap1A	Ras related GTPases
ERK	the extracellular signal-regulated kinase
VEGF	vascular endothelial growth factor
CXCR1/2	C-X-C chemokine receptors 1 and 2
ROS	reactive oxygen species
MMP-1	matrix metalloproteinase 1
PAR1	protease activated receptor 1
Human MCF7-PAR1 (clone N55)	stable high PAR1 expressing MCF7 clone 55 (note MCF-7 cells don not express PAR1)
PAR1-null	lack of PAR1 expression
Human MCF7-PAR1 (R310E)	stable cell line expression PAR1 with an R310E mutation that results in high expression of a PAR1 non-signaling receptor
NCR nu/nu mice	immuno-deficient mouse model
MMP-1 Inh	FN439 matrix metalloprotease 1 inhibitor
vWF	von Willebrand Factor
Human MDA-MB-231	PAR1 expressing breast cancer cell line
PZ-128	also known as P1pal-7 is the PAR1 antagonist pepducin
metastatic incidence	appearance of macroscopic tumor nodules at the surface of lung or using histologic analysis of lung sections
tumor incidence	number of tumors formed after subcutaneous implantation into mammary fat pads
TUNEL	terminal deoxynucleotidyl transferase-mediated dUTP nick end labeling
A549	human PAR1 expressing lung adenocarcinoma
LLC1	mouse Lewis lung carcinoma
LLC1-shPAR1	a short hairpin-RNA that silenced PAR1 expression
LLC1-shControl	control (shLuciferase)
*MMP1a-KO*	MMP1 knockout mouse
OVCAR-4 and SKOV-3	Human ovarian cell lines
Ascites formation	production of large volume of fluid in the peritoneal cavity
Intraperitoneal cavity	space between the abdominal wall and the surrounding interanal organs
Peritoneal carcinomatosis	mice were injected into the peritoneal cavity with ovarian cancer cells
FAK ERK1/2	focal adhesion kinase extracellular signal-regulated kinase 1 and 2

## References

[1] American Cancer Society (2018). Cancer Facts & Figures.

[2] Boire A., Covic L., Agarwal A., Jacques S., Sherifi S., Kuliopulos A. (2005). PAR1 is a matrix metalloprotease-1 receptor that promotes invasion and tumorigenesis of breast cancer cells. Cell.

[3] Kamath L., Meydani A., Foss F., Kuliopulos A. (2001). Signaling from Protease-activated Receptor-1 Inhibits Migration and Invasion of Breast Cancer Cells. Cancer Res..

[4] Yang E., Boire A., Agarwal A., Nguyen N., O’Callaghan K., Tu P., Covic L. (2009). Blockade of PAR1 signaling with cell-penetrating pepducins inhibits Akt survival pathways in breast cancer cells and suppresses tumor survival and metastasis. Cancer Res..

[5] Cisowski J., O’Callaghan K., Kuliopulos A., Yang J., Nguyen N., Deng Q., Agarwal A. (2011). Targeting protease-activated receptor-1 with cell-penetrating pepducins in lung cancer. Am. J. Pathol..

[6] Agarwal A., Covic L., Sevigny L.M., Kaneider N.C., Lazarides K., Azabdaftari G., Kuliopulos A. (2008). Targeting a metalloprotease-PAR1 signaling system with cell-penetrating pepducins inhibits angiogenesis, ascites, and progression of ovarian cancer. Mol. Cancer Ther..

[7] Agarwal A., Tressel S.L., Kaimal R., Balla M., Lam F.H., Covic L., Kuliopulos A. (2010). Identification of a metalloprotease-chemokine signaling system in the ovarian cancer microenvironment: Implications for antiangiogenic therapy. Cancer Res..

[8] Li M., Jiang C., Ye L., Wang S., Zhang H., Liu J., Hong H. (2017). The Role of Na+/Ca2+ Exchanger 1 in Maintaining Ductus Arteriosus Patency. Sci. Rep..

[9] Covic L., Gresser A.L., Talavera J., Swift S., Kuliopulos A. (2002). Activation and inhibition of G protein-coupled receptors by cell-penetrating membrane-tethered peptides. Proc. Natl. Acad. Sci. USA.

[10] O’Callaghan K., Kuliopulos A., Covic L. (2012). Turning receptors on and off with intracellular pepducins: New insights into G-protein-coupled receptor drug development. J. Biol. Chem..

[11] Gurbel P.A., Kuliopulos A., Tantry U.S. (2015). G-protein-coupled receptors signaling pathways in new antiplatelet drug development. Arterioscler. Thromb. Vas. Biol..

[12] Zhang P., Gruber A., Kasuda S., Kimmelstiel C., O’Callaghan K., Cox D.H., Kuliopulos A. (2012). Suppression of arterial thrombosis without affecting hemostatic parameters with a cell-penetrating PAR1 pepducin. Circulation.

[13] Gurbel P.A., Bliden K.P., Turner S.E., Tantry U.S., Gesheff M.G., Barr T.P., Kuliopulos A. (2016). Cell-Penetrating Pepducin Therapy Targeting PAR1 in Subjects With Coronary Artery Disease. Arterioscler. Thromb. Vas. Biol..

[14] Hollenberg M.D., Compton S.J. (2002). Proteinase-Activated Receptors. Pharmacol. Rev..

[15] Vu T.-K.H., Hung D.T., Wheaton V.I., Coughlin S.R. (1991). Molecular Cloning of a Functional Thrombin Receptor Reveals a Novel Proteolytic Mechanism of Receptor Action. Cell.

[16] Kuliopulos A., Covic L., Seeley S.K., Sheridan P.J., Helin J., Costello C.E. (1999). Plasmin Desensitization of the PAR1 Thrombin Receptor: Kinetics, Sites of Truncation, and Implications for Thrombolytic Therapy. Biochemistry.

[17] Seeley S., Covic L., Jacques S.L., Sudmeier J., Baleja J.D., Kuliopulos A. (2003). Structural basis for thrombin activation of a protease-activated receptor: Inhibition of intramolecular liganding. Chem. Biol..

[18] Trivedi V., Boire A., Tchernychev B., Kaneider N.C., Leger A.J., O’Callaghan K., Kuliopulos A. (2009). Platelet matrix metalloprotease-1 mediates thrombogenesis by activating PAR1 at a cryptic ligand site. Cell.

[19] Austin K.M., Covic L., Kuliopulos A. (2012). Matrix metalloproteases and PAR1 activation. Blood.

[20] Nguyen N., Kuliopulos A., Graham R.A., Covic L. (2006). Tumor-derived Cyr61(CCN1) promotes stromal matrix metalloproteinase-1 production and protease-activated receptor 1-dependent migration of breast cancer cells. Cancer Res..

[21] Foley C.J., Luo C., O’Callaghan K., Hinds P.W., Covic L., Kuliopulos A. (2012). Matrix metalloprotease-1a promotes tumorigenesis and metastasis. J. Biol. Chem..

[22] Foley C.J., Fanjul-Fernandez M., Bohm A., Nguyen N., Agarwal A., Austin K., Kuliopulos A. (2013). Matrix metalloprotease 1a deficiency suppresses tumor growth and angiogenesis. Oncogene.

[23] Foley C.J., Kuliopulos A. (2014). Mouse matrix metalloprotease-1a (Mmp1a) gives new insight into MMP function. J. Cell. Physiol..

[24] Rutter J.L., Benbow U., Coon C.I., Brinckerhoff C.E. (1997). Cell-type specific regulation of human interstitial collagenase-1 gene expression by interleukin-1 beta (IL-1 beta) in human fibroblasts and BC-8701 breast cancer cells. J. Cell. Biochem..

[25] Fanjul-Fernandez M., Folgueras A.R., Fueyo A., Balbin M., Suarez M.F., Fernandez-Garcia M.S., López-Otín C. (2013). Matrix metalloproteinase Mmp-1a is dispensable for normal growth and fertility in mice and promotes lung cancer progression by modulating inflammatory responses. J. Biol. Chem..

[26] Austin K.M., Nguyen N., Javid G., Covic L., Kuliopulos A. (2013). Noncanonical matrix metalloprotease-1-protease-activated receptor-1 signaling triggers vascular smooth muscle cell dedifferentiation and arterial stenosis. J. Biol. Chem..

[27] Jaffre F., Friedman A.E., Hu Z., Mackman N., Blaxall B.C. (2012). Beta-adrenergic receptor stimulation transactivates protease-activated receptor 1 via matrix metalloproteinase 13 in cardiac cells. Circulation.

[28] Mendonsa A.M., VanSaun M.N., Ustione A., Piston D.W., Fingleton B.M., Gorden D.L. (2015). Host and tumor derived MMP13 regulate extravasation and establishment of colorectal metastases in the liver. Mol. Cancer.

[29] Chang H.J., Yang M.J., Yang Y.H., Hou M.F., Hsueh E.J., Lin S.R. (2009). MMP13 is potentially a new tumor marker for breast cancer diagnosis. Oncol. Rep..

[30] Mosnier L.O., Sinha R.K., Burnier L., Bouwens E.A., Griffin J.H. (2012). Biased agonism of protease-activated receptor 1 by activated protein C caused by noncanonical cleavage at Arg46. Blood.

[31] Zhao P., Metcalf M., Bunnett N.W. (2014). Biased signaling of protease-activated receptors. Front. Endocrinol..

[32] Cierna Z., Mego M., Janega P., Karaba M., Minarik G., Benca J., Pindak D. (2014). Matrix metalloproteinase 1 and circulating tumor cells in early breast cancer. BMC Cancer.

[33] Przybylowska K., Kluczna A., Zadrozny M., Krawczyk T., Kulig A., Rykala J., Blasiak J. (2006). Polymorphisms of the promoter regions of matrix metalloproteinases genes MMP-1 and MMP-9 in breast cancer. Breast Cancer Res. Treat..

[34] Van Lint P., Libert C. (2007). Chemokine and cytokine processing by matrix metalloproteinases and its effect on leukocyte migration and inflammation. J. Leukoc. Biol..

[35] Kessenbrock K., Plaks V., Werb Z. (2010). Matrix metalloproteinases: Regulators of the tumor microenvironment. Cell.

[36] Page-McCaw A., Ewald A.J., Werb Z. (2007). Matrix metalloproteinases and the regulation of tissue remodelling. Nat. Rev. Mol. Cell Biol..

[37] Zhang C., Srinivasan Y., Arlow D.H., Fung J.J., Palmer D., Zheng Y., Weis W.I. (2012). High-resolution crystal structure of human protease-activated receptor 1. Nature.

[38] Zhang P., Leger A.J., Baleja J.D., Rana R., Corlin T., Nguyen N., Kuliopulos A. (2015). Allosteric Activation of a G Protein-coupled Receptor with Cell-penetrating Receptor Mimetics. J. Biol. Chem..

[39] Zhang P., Covic L., Kuliopulos A. (2015). Pepducins and Other Lipidated Peptides as Mechanistic Probes and Therapeutics. Methods Mol. Biol..

[40] Swift S., Leger A., Talavera J., Zhang L., Bohm A., Kuliopulos A. (2006). The Role of the PAR1 Receptor 8th Helix in Signaling: The 7-8-1 receptor activation mechanism. J. Biol. Chem..

[41] Yang E., Cisowski J., Nguyen N., O’Callaghan K., Xu J., Agarwal A., Covic L. (2015). Dysregulated protease activated receptor 1 (PAR1) promotes metastatic phenotype in breast cancer through HMGA2. Oncogene.

[42] Zhong W., Chen S., Qin Y., Zhang H., Wang H., Meng J., Han J. (2017). Doxycycline inhibits breast cancer EMT and metastasis through PAR-1/NF-kappaB/miR-17/E.-cadherin pathway. Oncotarget.

[43] Wang Y., Liu J., Ying X., Lin P.C., Zhou B.P. (2016). Twist-mediated Epithelial-mesenchymal Transition Promotes Breast Tumor Cell Invasion via Inhibition of Hippo Pathway. Sci. Rep..

[44] Kancharla A., Maoz M., Jaber M., Agranovich D., Peretz T., Grisaru-Granovsky S., Bar-Shavit R. (2015). PH motifs in PAR1&2 endow breast cancer growth. Nat. Commun..

[45] Arakaki A.K., Pan W.A., Lin H., Trejo J. (2018). The alpha-arrestin ARRDC3 suppresses breast carcinoma invasion by regulating G protein-coupled receptor lysosomal sorting and signaling. J. Biol. Chem..

[46] Pang L., Li J.F., Su L., Zang M., Fan Z., Yu B., Liu B. (2018). ALEX1, a novel tumor suppressor gene, inhibits gastric cancer metastasis via the PAR-1/Rho GTPase signaling pathway. J. Gastroenterol..

[47] Hatziapostolou M., Koukos G., Polytarchou C., Kottakis F., Serebrennikova O., Kuliopulos A., Tsichlis P.N. (2011). Tumor progression locus 2 mediates signal-induced increases in cytoplasmic calcium and cell migration. Sci. Signal..

[48] Sayyah J., Bartakova A., Nogal N., Quilliam L.A., Stupack D.G., Brown J.H. (2014). The Ras-related protein, Rap1A, mediates thrombin-stimulated, integrin-dependent glioblastoma cell proliferation and tumor growth. J. Biol. Chem..

[49] Andrikopoulos P., Kieswich J., Harwood S.M., Baba A., Matsuda T., Barbeau O., Yaqoob M.M. (2015). Endothelial Angiogenesis and Barrier Function in Response to Thrombin Require Ca2+ Influx through the Na+/Ca2+ Exchanger. J. Biol. Chem..

[50] Juncker-Jensen A., Deryugina E.I., Rimann I., Zajac E., Kupriyanova T.A., Engelholm L.H., Quigley J.P. (2013). Tumor MMP-1 activates endothelial PAR1 to facilitate vascular intravasation and metastatic dissemination. Cancer Res..

[51] Adams G.N., Rosenfeldt L., Frederick M., Miller W., Waltz D., Kombrinck K., Palumbo J.S. (2015). Colon Cancer Growth and Dissemination Relies upon Thrombin, Stromal PAR-1, and Fibrinogen. Cancer Res..

[52] Wang T., Jiao J., Zhang H., Zhou W., Li Z., Han S., Yan W. (2017). TGF-beta induced PAR-1 expression promotes tumor progression and osteoclast differentiation in giant cell tumor of bone. Int. J. Cancer.

[53] Hernandez N.A., Correa E., Avila E.P., Vela T.A., Perez V.M. (2009). PAR1 is selectively over expressed in high grade breast cancer patients: A cohort study. J. Transl. Med..

[54] Batlle E., Sancho E., Franci C., Dominguez D., Monfar M., Baulida J., de Herreros A.G. (2000). The transcription factor snail is a repressor of E-cadherin gene expression in epithelial tumour cells. Nat. Cell Biol..

[55] Lamouille S., Xu J., Derynck R. (2014). Molecular mechanisms of epithelial-mesenchymal transition. Nat. Rev. Mol. Cell Biol..

[56] Peinado H., Olmeda D., Cano A. (2007). Snail, Zeb and bHLH factors in tumour progression: An alliance against the epithelial phenotype?. Nat. Rev. Cancer.

[57] Ferguson M., Henry P.A., Currie R.A. (2003). Histone deacetylase inhibition is associated with transcriptional repression of the Hmga2 gene. Nucleic Acids Res..

[58] Kumar M.S., Armenteros-Monterroso E., East P., Chakravorty P., Matthews N., Winslow M.M., Downward J. (2014). HMGA2 functions as a competing endogenous RNA to promote lung cancer progression. Nature.

[59] Sheen Y.S., Liao Y.H., Lin M.H., Chu C.Y., Ho B.Y., Hsieh M.C., Chiu H.C. (2015). IMP-3 promotes migration and invasion of melanoma cells by modulating the expression of HMGA2 and predicts poor prognosis in melanoma. J. Investig. Dermatol..

[60] Alam M., Ahmad R., Rajabi H., Kufe D. (2015). MUC1-C Induces the LIN28B-->LET-7-->HMGA2 Axis to Regulate Self-Renewal in NSCLC. Mol. Cancer Res. (MCR).

[61] Thuault S., Tan E.J., Peinado H., Cano A., Heldin C.H., Moustakas A. (2008). HMGA2 and Smads co-regulate SNAIL1 expression during induction of epithelial-to-mesenchymal transition. J. Biol. Chem..

[62] Puca L., Brou C. (2014). Alpha-arrestins—New players in Notch and GPCR signaling pathways in mammals. J. Cell Sci..

[63] Holinstat M., Preininger A.M., Milne S.B., Hudson W.J., Brown H.A., Hamm H.E. (2009). Irreversible platelet activation requires protease-activated receptor 1-mediated signaling to phosphatidylinositol phosphates. Mol. Pharmacol..

[64] Arora P., Cuevas B.D., Russo A., Johnson G.L., Trejo J. (2008). Persistent transactivation of EGFR and ErbB2/HER2 by protease-activated receptor-1 promotes breast carcinoma cell invasion. Oncogene.

[65] Cattaneo F., Guerra G., Parisi M., De Marinis M., Tafuri D., Cinelli M., Ammendola R. (2014). Cell-surface receptors transactivation mediated by g protein-coupled receptors. Int. J. Mol. Sci..

[66] Wang H., Zhang X. (2017). Magnetic Fields and Reactive Oxygen Species. Int. J. Mol. Sci..

[67] Tressel S.L., Kaneider N.C., Kasuda S., Foley C., Koukos G., Austin K., Kuliopulos A. (2011). A matrix metalloprotease-PAR1 system regulates vascular integrity, systemic inflammation and death in sepsis. EMBO Mol. Med..

[68] Kaneider N.C., Leger A.J., Agarwal A., Nguyen N., Perides G., Derian C., Kuliopulos A. (2007). ‘Role reversal’ for the receptor PAR1 in sepsis-induced vascular damage. Nat Immunol..

[69] Sevigny L.M., Zhang P., Bohm A., Lazarides K., Perides G., Covic L., Kuliopulos A. (2011). Interdicting protease-activated receptor-2-driven inflammation with cell-penetrating pepducins. Proc. Natl. Acad. Sci. USA.

[70] Miller J., Agarwal A., Devi L.A., Fontanini K., Hamilton J.A., Pin J.P., Hunt III S.W. (2009). Insider access: Pepducin symposium explores a new approach to GPCR modulation. Ann. N. Y. Acad. Sci..

[71] Kaneider N.C., Agarwal A., Leger A.J., Kuliopulos A. (2005). Reversing Systemic Inflammatory Response Syndrome with Chemokine Receptor Pepducins. Nat. Med..

[72] Kuliopulos A., Covic L. (2003). Blocking receptors on the inside: Pepducin-based intervention of PAR signaling and thrombosis. Life Sci..

[73] Dimond P., Carlson K., Bouvier M., Gerard C., Xu L., Covic L., Gardella T.J. (2011). G protein-coupled receptor modulation with pepducins: Moving closer to the clinic. Ann. N. Y. Acad. Sci..

[74] Covic L., Tchernychev B., Jacques S., Kuliopulos A., Langel U. (2007). Pharmacology and in vivo efficacy of pepducins in hemostasis and arterial thrombosis. Handbook of Cell-Penetrating Peptides.

[75] O’Callaghan K., Lee L., Nguyen N., Hsieh M.Y., Kaneider N.C., Klein A.K., Covic L. (2012). Targeting CXCR4 with cell-penetrating pepducins in lymphoma and lymphocytic leukemia. Blood..

[76] Tchernychev B., Ren Y., Sachdev P., Janz J.M., Haggis L., O’Shea A., Kazmi M.A. (2010). Discovery of a CXCR4 agonist pepducin that mobilizes bone marrow hematopoietic cells. Proc. Natl. Acad. Sci. USA.

[77] Janz J.M., Ren Y., Looby R., Kazmi M.A., Sachdev P., Grunbeck A., McMurry T. (2011). Direct interaction between an allosteric agonist pepducin and the chemokine receptor CXCR4. J. Am. Chem. Soc..

[78] Rana R., Huang T., Koukos G., Fletcher E.K., Turner S.E., Shearer A., Covic L. (2018). Noncanonical Matrix Metalloprotease 1-Protease-Activated Receptor 1 Signaling Drives Progression of Atherosclerosis. Arterioscler. Thromb. Vasc. Biol..

[79] Covic L., Singh C., Smith H., Kuliopulos A. (2002). Role of the PAR4 Thrombin Receptor in Stabilizing Platelet-Platelet Aggregates as revealed by a Patient with Hermansky-Pudlak Syndrome. Thromb. Haemost..

[80] Even-Ram S., Uziely B., Cohen P., Grisaru-Granovsky S., Maoz M., Ginzburg Y., Bar-Shavit R. (1998). Thrombin Receptor Overexpression in Malignant and Physiological Invasion Processes. Nat. Med..

[81] Even-Ram S.C., Maoz M., Pokroy E., Reich R., Katz B.-Z., Gutwein P., Bar-Shavit R. (2001). Tumor Cell Invasion Is Promoted by Activation of Protease Activated Receptor-1 in Cooperation with the alpha vbeta 5 Integrin. J. Biol. Chem..

[82] Whitehead I., Kirk H., Kay R. (1995). Expression Cloning of Oncogenes by Retroviral Transfer of cDNA Libraries. Mol. Cell. Biol..

[83] Martin C.B., Mahon G.M., Klinger M.B., Kay R.J., Symons M., Der C.J., Whitehead I.P. (2001). The thrombin receptor, PAR-1, causes transformation by activation of Rho-mediated signaling pathways. Oncogene.

[84] Yin Y.-J., Salah Z., Maoz M., Ram S.C.E., Ochayon S., Neufeld G., Bar-Shavit R. (2003). Oncogenic transformation induces tumor angiogenesis: A role for PAR1 activation. FASEB J..

[85] Ho M.Y., Mackey J.R. (2014). Presentation and management of docetaxel-related adverse effects in patients with breast cancer. Cancer Manag. Res..

[86] Ghio P., Cappia S., Selvaggi G., Novello S., Lausi P., Zecchina G., Scagliotti G.V. (2006). Prognostic role of protease-activated receptors 1 and 4 in resected stage IB non-small-cell lung cancer. Clin. Lung Cancer.

[87] Cohen M.H., Gootenberg J., Keegan P., Pazdur R. (2007). FDA drug approval summary: Bevacizumab (Avastin) plus Carboplatin and Paclitaxel as first-line treatment of advanced/metastatic recurrent nonsquamous non-small cell lung cancer. Oncologist.

[88] Tricoci P., Huang Z., Held C., Moliterno D.J., Armstrong P.W., Van de Werf F., Lokhnygina Y. (2012). Thrombin-receptor antagonist vorapaxar in acute coronary syndromes. N. Engl. J. Med..

[89] Morrow D.A., Braunwald E., Bonaca M.P., Ameriso S.F., Dalby A.J., Fish F.P., Ophuis A.O. (2012). Vorapaxar in the Secondary Prevention of Atherothrombotic Events. N. Engl. J. Med..

[90] Valgimigli M., Costa F., Lokhnygina Y., Clare R.M., Wallentin L., Moliterno D.J., Van de Werf F. (2017). Trade-off of myocardial infarction vs. bleeding types on mortality after acute coronary syndrome: Lessons from the Thrombin Receptor Antagonist for Clinical Event Reduction in Acute Coronary Syndrome (TRACER) randomized trial. Eur. Heart J..

[91] O’Donoghue M.L., Bhatt D.L., Wiviott S.D., Goodman S.G., Fitzgerald D.J., Angiolillo D.J., Guetta V. (2011). Safety and tolerability of atopaxar in the treatment of patients with acute coronary syndromes: The lessons from antagonizing the cellular effects of Thrombin-Acute Coronary Syndromes Trial. Circulation.

[92] Costopoulos C., Niespialowska-Steuden M., Kukreja N., Gorog D.A. (2013). Novel oral anticoagulants in acute coronary syndrome. Int. J. Cardiol..

[93] O’Donoghue M.L., Bhatt D.L., Flather M.D., Goto S., Angiolillo D.J., Goodman S.G., Kobayashi H. (2012). Atopaxar and its effects on markers of platelet activation and inflammation: Results from the LANCELOT CAD program. J. Thromb. Thrombolysis.

[94] Wiviott S.D., Flather M.D., O’Donoghue M.L., Goto S., Fitzgerald D.J., Cura F., Angiolillo D.J. (2011). Randomized Trial of Atopaxar in the Treatment of Patients With Coronary Artery Disease: The Lessons From Antagonizing the Cellular Effect of Thrombin—Coronary Artery Disease Trial. Circulation.

